# Bilateral Successive Testicular Tumors With Different Cell Types

**DOI:** 10.4021/wjon342w

**Published:** 2011-12-19

**Authors:** Mathew K. George, Pinky Baghi

**Affiliations:** a Senior Consultant Oncologist, Tamworth Rural Referral hospital, Tamworth, New South Wales, Australia; b Advanced Trainee Medical Oncology, Tamworth Rural Referral hospital, Tamworth, New South Wales, Australia

**Keywords:** Testicular tumor, Cell type, Germ cell

## Abstract

We are presenting the case of a young man who had bilateral successive testicular tumors with different cell types. The time interval between the two tumors was about 6 years. This underlines the importance of recognising patients with germ cell tumors who are at risk of developing a second tumor and requires a long term follow up.

## Introduction

Testicular cancer although it accounts for about 1 percent of all cancers in men, is the most common solid malignancy affecting males between the ages of 15 and 35.

A small percentage of men with testicular cancer will have a second testicular cancer, and the incidence of bilateral tumors at presentation is between 1 and 5 percent. The largest study analysed 29,515 cases of testicular cancer in men under the age of 55 reported to the United States National Cancer Institute Surveillance, Epidemiology and End Results (SEER) program between 1973 and 2001 [1]. The 15-year cumulative risk of a contra lateral (metachronous) testicular cancer was 1.9 percent.

We report here a case of a man who had successive bilateral testicular tumors with different cell types-seminoma of left testis 2002 and later non-seminoma of right testis carcinoma in 2009.

## Case Report

Patient was diagnosed in 2002 as left testicular carcinoma-seminoma stage 1. He had left orchidectomy and para aortic radiation. There was no evidence of metastasis and was on follow up since then.

He presented again in 2009 with lump in right testis. His tumor markers were negative. Ultasound showed highly suspicious abnormality with 2 cm mass in midzone and 1.4 cm mass in lower pole, both suggestive of malignancy. On CT, there were no enlarged lymph nodes in the retro peritoneum and pelvic structures were normally defined.

He underwent right inguinal orchidectomy. Pathology report revealed mixed germ cell tumor consisting of embryonal carcinoma and seminoma with extensive vascular involvement by embryonal carcinoma. Later CT scans in 2009 showed small aortocaval lymph nodes.

The absence of direct organ extension or demonstrable metastatic lymphatic or vascular involvement points to the primary origin of these lesions.

Patient was treated with BEP for this stage 1B non seminoma germ cell tumor. Currently patient is on regular follow up with tumor markers and scans. He is asymptomatic with no evidence of any metastasis.

## Discussion

A similar case of bilateral successive testicular cancer of different cell types (seminoma and embryonal carcinoma) was presented by B. Mittal et al. [2]. The time interval between the two tumors was 30 months.

In 1983, Bach et al reviewed 396 cases of bilateral testicular tumors reported in the literature through 1979. In their study of 18 cases of bilateral testicular tumors the tumors were the same histologically in 5 patients (seminoma in 4 and nonseminoma in 1), different in 6 and malignant lymphomas in [3].

Hirata Y et al reported an 18-year-old man who presented with right scrotal swelling. Right radical orchiectomy was done followed by chemotherapy, retroperitoneal lymph node. Five years after the treatment, the patient found a contra lateral scrotal mass. Serum.ALPHA.-fetoprotein level was increased, and ultrasonography revealed a 3.0 cm solid mass on the lower margin of the left testis. Left radical orchiectomy done and histopathological diagnosis was mixed type of immature teratoma, embryonal carcinoma, yolk sac tumor and choriocarcinoma [4].

In a report in 2002, the CheM presented a study involving 2431 patients with testicular germ cell tumors who were treated at The University of Texas M. D. Anderson Cancer Center over a 20-year period (Between 1978 and 1999). Among these, 24 patients (1%) were found to have bilateral germ cell tumors. The incidence was 1.8% (14 of 776 patients) in patients with seminoma and 0.6% (10 of 1655 patients) in patients with nonseminomatous germ cell tumors. Patients with seminoma who were age ≤ 30 years at the time of initial diagnosis had a higher incidence of bilateral tumors compared with older men. Twenty of 24 patients with bilateral germ cell tumors had metachronous tumor, and 4 patients had synchronous tumors. Among the patients with metachronous tumors, 70% of second tumors occurred within 5 years. Patients in the second or third decade of life who presented with seminomas as their initial tumor were more likely to develop a second germ cell tumor compared with patients in the fourth or fifth decade of life [5].

So this is important for clinical management of patients and identifying a population of patients with germ cell tumors who are at risk of developing a second tumor and requires a long term follow up.
